# Diagnosis and Treatment of Snapping Scapula Syndrome: A Scoping Review

**DOI:** 10.1177/19417381211029211

**Published:** 2021-07-09

**Authors:** Hassan Baldawi, Kyle Gouveia, Chetan Gohal, Latifah Almana, Ryan Paul, Bashar Alolabi, Jaydeep Moro, Moin Khan

**Keywords:** scapulothoracic bursitis, scapular disorders, shoulder rehabilitation

## Abstract

**Context:**

Snapping scapula syndrome (SSS) is commonly misdiagnosed and underreported due to lack of awareness.

**Objective:**

This scoping review aims to summarize the current evidence related to SSS diagnosis and treatment to aid clinicians in managing the condition more effectively.

**Data Sources:**

PubMed, Medline, and Embase databases were searched for studies related to the etiology, diagnosis, or treatment of SSS (database inception to March 2020).

**Study Selection:**

Databases were searched for available studies related to the etiology, diagnosis, or treatment of SSS.

**Study Design:**

A scoping review study design was selected to explore the breadth of knowledge in the literature regarding SSS diagnosis and treatment.

**Level of Evidence:**

Level 4.

**Data Extraction:**

Primary outcomes abstraction included accuracy of diagnostic tests, functional outcomes, and pain relief associated with various nonoperative and operative treatment options for SSS.

**Results:**

A total of 1442 references were screened and 40 met the inclusion criteria. Studies commonly reported SSS as a clinical diagnosis and relied heavily on a focused history and physical examination. The most common signs reported were medial scapular border tenderness, crepitus, and audible snapping. Three-dimensional computed tomography had high interrater reliability of 0.972, with a 100% success rate in identifying symptomatic incongruity of the scapular articular surface. Initial nonoperative treatment was reported as successful in most symptomatic patients, with improved visual analogue scale (VAS) scores (7.7 ± 0.5 pretreatment, to 2.4 ± 0.6). Persistently symptomatic patients underwent surgical intervention most commonly involving bursectomy, superomedial angle resection, or partial scapulectomy. High satisfaction rates of surgery were reported in VAS (6.9 ± 0.7 to 1.9 ± 0.9), American Shoulder and Elbow Surgeons scores (50.3 ± 12.2 to 80.6 ± 14.9), and mean simple shoulder test scores (5.6 ± 1.0 to 10.2 ± 1.1).

**Conclusion:**

Focused history and physical examination is the most crucial initial step in the diagnostic process, with supplemental imaging used to assess for structural etiologies when nonoperative management fails. Nonoperative management is as effective as surgical management in pain relief and is advised for 3 to 6 months before operative treatment.

Snapping scapula syndrome (SSS), washboard syndrome, or scapulothoracic crepitobursitis are all terms that were initially described by Boinet in 1867, where a 19-year-old male patient is described complaining of crepitus and discomfort with scapular movement.^5^ This syndrome is a commonly misdiagnosed and underreported condition of the scapulothoracic joint usually associated with painful crepitus and shoulder joint dysfunction when attempting overhead motion.^29,48^ The scapulothoracic joint is unique, as it lacks true synovial articulation and is dynamically controlled through surrounding muscular contractions. The scapula glides on the posterior thorax covered with muscle layers rather than a cartilaginous surface. This movement is facilitated by the infraserratus, subserratus, and trapezoid bursae between the 2 articulating surfaces, which is necessary for scapulothoracic motion.^8,12^ SSS is characterized by audible crepitus or snapping sensation associated with pain on overhead arm raising. These symptoms are created by the excessive friction between the scapula and the thorax with soft tissue (bursa, tendon, or muscle) entrapped between them.^20,21^

Common SSS etiologies include incongruency between the scapula and convex thorax resulting from space-occupying lesions (eg, osteochondromas), Luschka’s tubercle, bursitis secondary to acute or repetitive traumas, and increased anterior angulation of the superomedial scapular edge secondary to scapular muscle imbalances and chronic kyphotic posture.^3,11,51^ Regardless of the cause, any factor resulting in disturbance of the physiological scapulothoracic wall interface can increase the predisposition to SSS.^20^

Scapular biomechanical and kinetic chain dysfunction can produce several related conditions with varying severity. Scapular dyskinesis (SD) is defined as an altered scapular position and motion about the thorax. This altered scapular position can result in an abnormal scapulothoracic articulation leading to bursitis, which can exacerbate into SSS when crepitus is present.^51^ This constellation of related conditions can also result in SICK scapula syndrome (scapular malposition, inferomedial border prominence, anterior coracoid pain, and SD), which is an extreme form of SD and a pathology associated with the throwing shoulder.^6^

Diagnosis of SSS is challenging and includes a physical examination and advanced imaging such as magnetic resonance imaging (MRI) and/or computed tomography (CT) to assess for potential bony or soft tissue etiologies of SSS. Diagnostic local anesthetic or steroid injections administered at the point of maximum tenderness are used to identify possible bursitis, as symptomatic relief can confirm a bursitis diagnosis and the affected bursa’s location. Without aggressive space-occupying lesions, nonoperative treatment is initiated through rehabilitation exercises, activity modification, and pain management. If nonoperative management has proven ineffective, open or arthroscopic scapular superomedial resection and bursectomy is considered.^1,12,21,29,36^

SSS is commonly unidentified and can thus go untreated. This review evaluates the available literature to provide clinicians with an evidence-based summary on the diagnosis and management of this condition with the aim to minimize its misdiagnosis in the future.

## Methods

### Search Strategy

This scoping review was synthesized according to the Preferred Reporting Items for Systematic Reviews and Meta-Analysis for Scoping Reviews (PRISMA-ScR),^49^ starting with a research question that was developed using population, concept, and context methodology. A common search strategy was employed to search for all publications relevant to our topic using 3 electronic databases: Medline, PubMed, and Embase, from inception to March 2020. Common terms were searched across all databases that are typical snapping scapula features and associated presentations (eg, snapping scapula, scapular malposition, scapulothoracic bursitis, scapular pain). The full search strategy is available in Appendix Table 1 (available in the online version of this article). References were hand searched for any additional articles that could be included.

### Study Screening

The titles, abstracts, and full texts were screened by 2 independent reviewers in duplicate, using the online software Rayyan QCRI (2010, Qatar Computing Research Institute, Doha, Qatar). Disagreements during the title and abstract stage were carried forward to the next screening stage, and any disagreements at the full-text stage were discussed and resolved by a senior author.

### Study Eligibility

To be included, publications needed to be investigating diagnosis, etiology, treatment, or rehabilitation of SSS or relevant predisposing conditions. All study designs were included in this review, with the exception of case reports and publications lacking primary data (ie, systematic reviews and meta-analyses, editorial commentaries, opinion pieces). Cadaveric, anatomic, and surgical technique studies were excluded from this review.

### Data Abstraction

Demographic data (eg, age, gender, body mass index), etiological data (eg, type of overhead activity, traumatic, oncological), intervention outcomes (eg, pain, range of motion [ROM], self-reported functional scales), and diagnostic accuracy (eg, interrater reliability [IRR], intrarater reliability, predictive value) were abstracted into an online collaborative spreadsheet (Google) by 2 independent reviewers. Discrepancy in data collection was reoffered to a senior author to resolve.

### Methodological Quality Assessment

The Methodological Index for Non-Randomized Studies (MINORS) was used to assess the quality of non-randomized studies in this publication.^44^ The Cochrane Risk of Bias assessment was used for the included randomized controlled trials (RCTs).^14^

### Assessment of Agreement

Agreement between reviewers was evaluated using the Cohen kappa statistic (κ) at all screening stages. Agreement was classified a priori as follows: κ of 0.81 to 0.99 was considered nearly perfect agreement, κ of 0.61 to 0.80 was substantial agreement, κ of 0.41 to 0.60 was moderate agreement, 0.21 to 0.40 fair agreement, and a κ of 0.20 or less was considered slight agreement.^4^

### Statistical Analysis

A descriptive analysis was carried out for the included studies. Studies were categorized into diagnosis, treatment (both operative and nonoperative), and posttraumatic SD. Descriptive statistics are presented in absolute frequencies with percentages or weighted means with measures of variance where applicable.

## Results

### Literature Search

**Figure 1.**
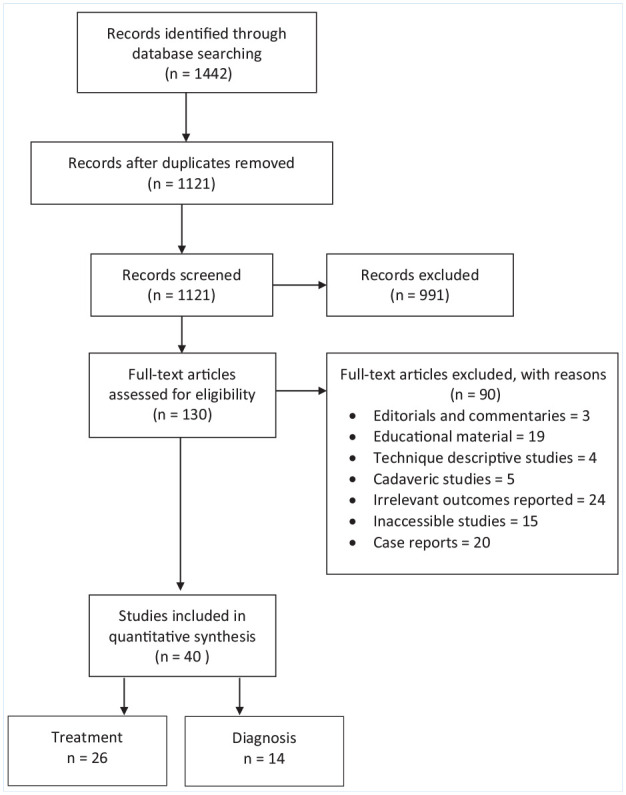
Preferred Reporting Items for Systematic Reviews and Meta-Analysis (PRISMA) flowchart.

The initial search yielded 1442 studies. After the removal of duplicates, 1121 studies remained. After systematic screening and assessment of eligibility, 40 studies were included in this review (Figure 1). Agreement between the reviewers was moderate at the title and abstract stage (κ = 0.48; 95% CI 0.38-0.58) and perfect at the full text stage (κ = 1.00).

Of the 40 included studies, 26 investigated SSS treatment, and 14 were related to diagnosis (including 3 studies that specifically examined the posttraumatic development of SD shown in Appendix Table 1, available online). Of the 26 studies examining SSS treatment, 4 were RCTs (level 1 evidence), 3 prospective cohort studies (level 2 evidence), 1 retrospective cohort study (level 3 evidence), and 18 case series (level 4 evidence). Higher level evidence (level 1 or 2) accounted for 75% (6 of 8) of nonoperative studies, but only 5.6% (1 of 18) of surgical treatment studies. For the 22 nonrandomized treatment studies, the mean MINORS scores were 10 and 16 for noncomparative and comparative studies, respectively. Risk of bias in the 4 RCTs is reported in Appendix Table 2 (available online).

### Study Characteristics

A total of 1138 patients were included in this review. The mean age in the study was 31.8 years (range, 6-81 years), with 60.3% (562 of 932) being men. For the 642 patients in treatment studies, the mean follow-up was 31.9 months (range, 2-420 months) from treatment. Of the 26 treatment studies, 69.2% (18 studies, 393 patients) involved surgical treatment, 15.4% (4 studies, 113 patients) involved exercise or rehabilitation, 7.7% (2 studies, 58 patients) involved injections, and 3.8% (1 study, 35 patients) involved extracorporeal shock wave therapy (ESWT). The 1 additional study (43 patients) compared ESWT with corticosteroid injection (CSI).

### Diagnosis

Eleven studies (404 patients) evaluated the diagnosis of SSS and SD as shown in Appendix Table 3 (available online). Three studies reported on diagnostic imaging, while 8 used clinical examination. The use of 3-dimensional computed tomography (3D-CT) had a very high IRR of 0.972,^38^ with 1 study having a 100% success rate (26 of 26) in identifying bony scapular incongruency, compared with 27% (7 of 26) from plain radiographs.^34^ Similarly, axial plane MRI evaluating scapular morphology found that anterior angulation of the medial scapula to be associated with SSS.^46^ Of the 8 studies using clinical assessment, the intraclass correlation coefficients (ICCs) for IRRs were reported in 3 studies, and were >0.80 in all 3.^29,37,43^ In the 2 studies reporting interrater agreement, this ranged from 83% to 86%.^9,16^ One additional study reported that clinical observation was only appropriate for diagnosing type I SD,^31^ and 1 found that while multiple tests were reliable, they carried questionable clinical importance.^36^ Last, in a review on SSS diagnosis, medial scapular border tenderness, palpable crepitus, and audible snapping were the most common clinical signs found.^40^

### Nonoperative Treatment

Nonoperative treatment for SSS was analyzed in 8 studies (249 patients), as shown in Appendix Table 4 (available online), with a mean age of 37.3 years and a mean follow-up of 5.9 months (range, 2.5-12 months). Visual analogue scale (VAS) pain scores were reported by 4 studies (2 investigated the use of ESWT, and 2 reported on CSI)^1,2,7,8^ and improved from a mean of 7.7 ± 0.5 pretreatment to 2.4 ± 0.6 at the latest follow-up. While the minimal clinically important difference (MCID) was not reported by the aforementioned studies, the MCID for VAS has been previously reported to be 3, resulting in clinically significant reported pain reduction.^20^ In the 2 studies utilizing CSI into the subscapularis bursa, significant decreases in VAS pain scores were seen at the latest follow-up of 3 months, without serious adverse events.^7,8^ In a study comparing injections to ESWT, both were initially effective; however, the ESWT group had lower VAS scores on later follow-ups (3 and 6 months). One study further compared low- and middle-energy ESWT, with middle-energy ESWT resulting in better pain scores at 6 months and 1 year.^1^ CSI was found to provide less pain relief when compared with ESWT. CSI achieved a mean of 37-point reduction in VAS score on a 100-point scale after 6 months of treatment, compared with a mean of 60-point reduction in VAS score 6 months after ESWT.^1,2^ Another study suggested that optimal VAS score reduction and symptom improvement is offered by middle-energy ESWT compared with low-energy ESWT (level 3 evidence), especially with long-term results.^1^

All 4 studies using exercise or rehabilitation found clinical improvement posttreatment.^10,12,30,39^ Rehabilitation aimed at restoring scapular muscle balance, decreasing pain, and improving rotator cuff strength.^30^ Additionally, 18 of 23 patients with type III acromioclavicular (AC) joint dislocation and SD who adopted a rehabilitation protocol had no dyskinesis at 12 months and thus no SSS.^12^ De Amorim et al^10^ compared segmental stretching exercises with global postural reeducation (GPR) (stretching shortened muscles while enhancing antagonistic contraction to avoid postural asymmetry along muscle group chains) and both improved SSS symptoms; however, GPR was superior when it came to pain and quality of life improvement. Also, Pekyavas et al^39^ compared home exercise to virtual reality (VR) exergaming, while both improved pain, VR exergaming resulted in better performance on clinical tests for dyskinesis.

### Operative Treatment

Eighteen studies (393 patients) reported on surgical treatment, with a mean age of 39.1 years and mean follow-up of 47.7 months (range, 3-420 months), as shown in Appendix Table 4 (available oline). Scapulothoracic bursectomy and superomedial scapular resection were the most frequently performed surgical procedures. One study managed SSS with pectoralis minor tendon release.^41^ Nine of the 18 studies specifically reported indications for surgical management, and all required a painful snapping scapula and failure of nonoperative treatment.

Five studies^13,23,35,41,47^ reported VAS pain scores on a 0 to 10 scale. All improved postoperatively, from a mean of 6.9 ± 0.7 to a mean of 1.9 ± 0.9. These studies investigated a combination of open and endoscopic scapulothoracic bursectomy, pectoralis minor tendon release, and partial scapular resection. The most commonly reported functional outcome scores were the American Shoulder and Elbow Surgeons (ASES) score (6 studies),^23,25,26,35,41,47^ and the simple shoulder test (SST, 4 studies).^23,27,35,47^ Mean ASES scores improved from 50.3 ± 12.2 to 80.6 ± 14.9 postoperatively, and SST scores from 5.6 ± 1.0 to 10.2 ± 1.1. Notably, 4 of 6 reporting studies met the MCID of 27.1 for the ASES score and 2 of 4 studies met the MCID of 4.3 for the SST.^48^ Additionally, 3 studies reported return to sport postoperatively. In these studies, 82% (18 of 22) returned to any level of sport, and 64% (14 of 22) returned to their preinjury level. Eleven studies (185 patients) reported postoperative complications. There was a total of 12 complications reported, with a reported pooled complication rate of 6.5% (12 of 185). Eight were reported as failed surgical treatments,^26^ and with others being wound infection (n = 1), hematoma (n = 1), long thoracic nerve injury (n = 1), and a procedure that was abandoned intraoperatively because of excessive swelling (n = 1).

Finally, 1 study (24 patients, level 3 evidence) compared open superomedial scapular resection to nonoperative management for milder SSS presentations.^50^ The authors concluded no significant difference between operative and nonoperative SSS management outcome in their series, with the operative group presenting with more pain at baseline. Because of the nonrandomized nature of this study and difference in symptom severity of the 2 groups preoperatively, it was cautioned that the study could not definitively conclude that nonoperative management is superior.^50^

## Discussion

**Figure 2.**
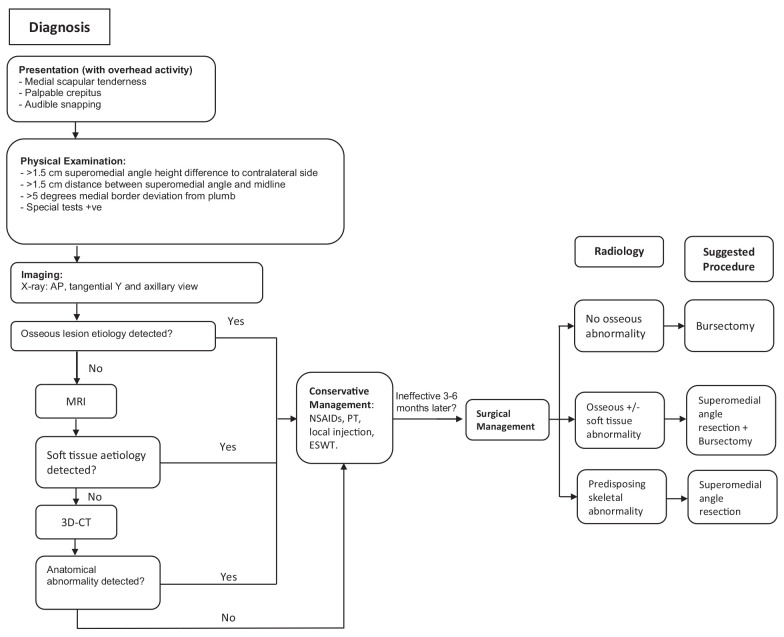
Diagnosis and management flowchart. 3D-CT, three-dimensional computed tomography; AP; ESWT, extracorporeal shock wave therapy; MRI, magnetic resonance imaging; NSAIDs, non-steroidal anti-inflammatory drugs; PT, physical therapy.

SSS continues to be an underrecognized source of shoulder morbidity. This scoping review summarizes the best available literature on the diagnosis and management of SSS and provides a treatment algorithm for patients with this condition (Figure 2).

### Diagnosis

Clinical observational studies in this review (n = 3) reported that diagnosis of SSS based on clinical assessment alone achieved ICC > 0.80 in 3 studies. Furthermore, 2 studies reported high interrater agreement for diagnosis based on history and physical examination (83%-86%).^11,16^ This suggests that diagnosis based on clinical assessment is quite reliable; however, this is contingent on being aware of the relevant history and examination findings.

Among patients diagnosed with SSS, this review found the most common clinical signs to be medial scapular border tenderness, palpable crepitus on shoulder movement, and audible snapping.^40^ Activity-related pain may vary from discomfort to disabling.^18,40,51^ However, scapulothoracic crepitus alone is commonly reported in asymptomatic patients and does not necessitate treatment.^51^

Physical examination should evaluate for spinal deformities, palpable crepitus, point tenderness, and scapular winging. Kyphoscoliosis can decrease scapulothoracic congruity, causing snapping scapula. Symmetry should be assessed to rule out periscapular muscle atrophy. Neurological assessment to rule out referred pain is essential. Scapular winging is a common presentation in patients with scapulothoracic bursitis or snapping scapula, which can occur from long thoracic nerve injury and dysfunction of the serratus anterior muscles.^30,36,51^ Deep palpation under the medial scapular border is achieved by putting the arm in “chicken wing” position (internally rotating the shoulder with dorsum of the hand placed over lumbosacral junction) as it helps tilt the scapula laterally.^33^ Passive and active ROM of the shoulder should be assessed to identify movement restrictions and overhead movement-related symptoms. Direct visualization of scapular movement with shoulder abduction is key to identify dyskinesis. Scapular crepitations can be further accentuated by applying posterior-anterior pressure during ROM.^32^

Scapular symmetry is one of the main features reported in the literature. Important comparisons include the height difference of the superomedial scapular angle of the 2 scapulae, the difference in distance of the superomedial angle from midline, and the difference in angular degrees of the medial scapular border from the plumb line, with 1.5 cm or 5° asymmetry being the threshold of abnormality in each of the measurements above.^6^ This asymmetry may also be the result of medial scapular muscle tears or dysfunction, leading to the development of SD and consequentially, SSS. This highlights the importance of assessing the muscle strength of periscapular muscles posteriorly and pectoralis minor anteriorly.^17^ In suspected cases of SSS, Miachiro et al^31^ used fatigue-inducing exercise protocol to fatigue periscapular muscles, exaggerating their dysfunction and increasing the specificity of the clinical examination.

One study in this review suggested that physical examination can only accurately diagnose type I SD (type I—prominence of inferomedial scapular angle; type II—prominence of medial scapular border; type III—superior scapular border elevation with anterior displacement), possibly since this type has the greatest difference between the maximum and minimum anterior tilt of the scapula compared with types II and III.^31^ Nonetheless, physical examination remains critical in the diagnostic pathway and to guide next steps.

Although the diagnosis of SSS may be achieved with the appropriate clinical assessment described earlier, determining the underlying etiology may still require further imaging and work-up. While plain film radiographs are the traditional first choice because of their ease of access and low associated morbidity, Moes et al^34^ reported them unreliable for definite diagnosis with only 26.9% detection of scapular bony incongruity, compared with 70% detection using CT and 100% detection achieved by 3D-CT.^34^ Park et al^38^ confirmed these findings, reporting excellent IRR (0.972) with the use of 3D-CT for symptomatic patients with bony scapular incongruity. The authors recommended the use of 3D-CT to precisely determine the type of SD, as the thick layer of soft tissue overlaying the scapula can make it difficult to determine the type of SD using observational methods alone. SD is a major predisposing factor for SSS, and diagnosing the type of SD determines the structural abnormalities involved and helps guide management.^38^

Despite the excellent IRR of CT for the diagnosis of bony scapulothoracic incongruity, it has demonstrated poor correlation to clinical findings in the setting of nonskeletal etiologies of SSS, such as scapulothoracic bursitis or other soft tissue precipitants.^51^ Limitations like radiation exposure, cost, and poor detection of soft tissue etiologies render CT unsuitable for routine SSS diagnosis. CT with or without 3D optimization could be beneficial in further characterizing space-occupying skeletal lesions in scapulothoracic space and skeletal incongruity after plain film detection.^38,51^

MRI continues to be the most useful diagnostic method in detection of soft tissue etiologies of SSS. MRI can accurately outline the nature and heterogeneity of soft tissue lesions, providing additional information to treat according to the specific pathology. Therefore, MRI use is recommended in investigating scapulothoracic soft tissue and space-occupying lesions as potential etiologies of SSS, when nonoperative treatment fails after clinical diagnosis.^15^

A summarized diagnosis and treatment algorithm for SSS is shown in Figure 2. From a diagnostic perspective, patients with scapular pain and/or crepitus with overhead movement should be assessed for SSS. If a diagnosis of SSS is achieved from clinical assessment, a nonoperative treatment plan should be initiated. Radiography can be initially used to detect any skeletal abnormality, which can be further characterized by CT if found. Alternatively, MRI is performed if soft tissue etiology is suspected (Figure 2).^19,21,34^ Cross-sectional imaging should be reserved for patients with osseous findings on radiography or those who failed nonoperative treatment and require further investigating.

### Management

Nonoperative treatment of SSS can be as effective as surgical options for the majority of patients and underlying etiologies.^24,50^ In this review, 4 studies (2 CSI and 2 ESWT therapy) found improved VAS scores from a mean of 7.7 ± 0.5 pretreatment to 2.4 ± 0.6 at the latest follow-up, which is comparable to the the 5 operative management studies reporting VAS improvement postoperatively, from a mean of 6.9 ± 0.7 to a mean of 1.9 ± 0.9.^1,2,7,8,13,23,35,41,47^ Notably, this improvement in VAS is greater than the MCID of 3 for both groups. Only 1 comparative study was identified between open superomedial scapular resection to nonoperative management and no significant difference was found between groups in the low-power study. Operative management should be reserved for patients who fail a 3- to 6-month trial of nonoperative modalities.

Physiotherapy and rehabilitation are the mainstay in nonoperative management of SSS and aim to address altered posture, scapular winging, or scapulothoracic dyskinesis.^24^ Scapular malposition can lead to abnormal force distribution throughout the shoulder joint, resulting in abnormal shoulder kinematics and problems with motion.^5^ Controlled scapular position on the thorax is essential for optimal shoulder function, providing maximum force to the rotator cuff muscles while contracting.^45^ Thus, the direction of the rehabilitation plan will depend on factors causing the snapping scapula. All exercise and rehabilitation publications in this review reported improvement in clinical parameters, decreased VAS scores, and improved rotator cuff muscle strength after restoration of scapular muscle balance.^10,12,30,39^

A number of studies also reported on the effectiveness of CSI as initial nonoperative treatment,^7^ which can be particularly useful as a diagnostic tool differentiating between scapular superomedial angle pathology and scapulothoracic bursitis in patients with superomedial angle pain.^35^ ESWT is another nonoperative modality examined in this review. A study comparing injections to ESWT found both to be initially effective; however, more pain relief was achieved with ESWT at 3 and 6 months.^1^ Both ESWT and injections can be utilized as adjuncts to the rehabilitation program.

Available literature suggests surgical treatment is warranted after failure of 3 to 6 months of nonoperative management for patients with symptomatic SSS.^28,33,51^ The most commonly performed surgical procedures are scapulothoracic bursectomy and superomedial scapular resection. All surgical studies reviewed reported pain and functional improvement postoperatively, with a mean VAS score improvement from 6.9 to 1.9, mean ASES scores improved from 50.3 ± 12.2 to 80.6 ± 14.9, and mean SST scores from 5.6 ± 1.0 to 10.2 ± 1.1.^13,23,25-27,35,41,47^

Identification of the underlying etiology for SSS is paramount as anatomic variations in scapular morphology can predispose to SSS, and nonoperative treatment may not be effective in these cases.^50^ In 13 cases examined by Lesprit et al,^22^ nonoperative treatment was 50% (5 of 10) effective in alleviating idiopathic SSS symptoms. On the other hand, when a skeletal abnormality was identified and treated, surgical treatment with superior angle or osteochondroma resection was reported to have good results in 7 of 8 patients.^21,35^

Arthroscopic or open scapulothoracic bursectomy is recommended for refractory patients who are symptomatic with no evidence of scapular skeletal abnormality on imaging. Bursectomy combined with partial scapulectomy is one of the most commonly performed procedures for SSS, aiming to remove soft tissue and skeletal precipitants.^35^ The choice of arthroscopic versus open or combined procedures largely depends on the surgeon’s experience and there is limited evidence comparing the 2 procedures. Arthroscopy offers improved cosmesis and earlier rehabilitation. Potential disadvantages of the arthroscopic approach include the risk of injury to neurovascular structures when penetrating the rhomboids, intraoperative swelling, and the inability to evaluate the potentially pathologic trapezoid bursa.^42^ Open technique in beach-chair position offers potential benefits such as excellent visualization of relevant structures, requiring less operative time and minimizing fluid extravasation to the ipsilateral shoulder.^18^ Regarding the arthroscopic technique, prone position with the affected arm being placed in extension and internal rotation (so-called chicken wing position) has been recommended, as it allows for excellent access to the entire scapula.^33^ In addition, surgical release of the pectoralis minor tendon has been reported to be effective when a tight pectoralis minor fails stretching and rehabilitation exercises.^41^

### Limitations

This review is limited by the low quality and heterogeneity of included studies. Rarity in SSS diagnosis resulted in underpowered studies with small sample sizes, limiting definitive conclusions. The outcomes of the variable treatment modalities and surgical options in this scoping review were treated homogenously to aggregate the results in a conclusive manner. This limits the ability to discriminate between different modalities and their individual impacts on the outcome scores. While RCTs were included, the majority of publications included were of level 3 or 4 evidence, which highlights the need to personalize these results to individual patients, and the need for further high quality RCTs to draw reliable conclusions.^44^

## Conclusion

High clinical suspicion for SSS is necessary in patients presenting with medial scapular border tenderness, palpable crepitus, and audible snapping. Focused history and physical examination are essential initial steps toward the diagnosis, with supplemental imaging to assess structural etiologies when nonoperative management fails. Nonoperative management of SSS in the form of analgesia, physiotherapy, local CSI, and/or ESWT should be initiated for 3 to 6 months before considering surgical management. Open or arthroscopic bursectomy with or without superomedial angle resection can then be carried out for refractory patients depending on the musculoskeletal pathology presented (Figure 2).

## Supplemental Material

sj-docx-1-sph-10.1177_19417381211029211 – Supplemental material for Diagnosis and Treatment of Snapping Scapula Syndrome: A Scoping Review Click here for additional data file. Supplemental material, sj-docx-1-sph-10.1177_19417381211029211 for Diagnosis and Treatment of Snapping Scapula Syndrome: A Scoping Review by Hassan Baldawi, Kyle Gouveia, Chetan Gohal, Latifah Almana, Ryan Paul, Bashar Alolabi, Jaydeep Moro and Moin Khan in Sports Health: A Multidisciplinary Approach
