# Supplementary material for: Diagnosis and Treatment of Snapping Scapula Syndrome: A Scoping Review
Source: Sports Health. 2021 Jul 9;14(3):389–96. doi: 10.1177/19417381211029211 (PMC9109590; doi:10.1177/19417381211029211)
Supplement: sj-docx-1-sph-10.1177_19417381211029211 – Supplemental material for Diagnosis and Treatment of Snapping Scapula Syndrome: A Scoping Review [file sj-docx-1-sph-10.1177_19417381211029211.docx]

**Appendix Table 1. Studies investigating diagnosis by clinical and radiological methods.**

| **Study** | **Study Participants (n)** | **Mean Age** | **Diagnostic Modality** | **Brief Description** | **Key Findings** |
| --- | --- | --- | --- | --- | --- |
| Christiansen et al. 2017 | 40 | 48.1 | Physical exam test | Utilized the scapular dyskinesis test (SDT). | The SDT had almost perfect reliability and agreement. However, those with scapular dyskinesis were no different when it came to functional outcomes. |
| Huang et al. 2015 | 60 | 22.5 ± 2.6 | Physical exam test | Visual based palpation method was used by two independent physiotherapists to classify scapular dyskinesis. | The visual-based palpation classification method for scapular dyskinesis had moderate to substantial interrater reliability. |
| Merolla et al. 2010 | 35 | 23 ± 4.5 | Physical exam test | Utilized the infraspinatus scapular retraction test (ISRT). | The ISRT had good reliability and easy reproducibility, making it a useful test to evaluate patients with infraspinatus weakness due to scapular dyskinesis. |
| Miachiro et al. 2014 | 26 | 22.8 ± 3.4 | Clinical observation | Investigated diagnosis of scapular dyskinesis by clinical observation by an expert physiotherapist. | Clinical observation alone was only considered appropriate in the diagnosis of Type I scapular dyskinesis. |
| Mozes et al. 1999 | 20 | 29 | 3D CT | 3D CT was used to evaluate scapular bony morphology. | 3D CT was more effective than plain radiographs or CT in identifying scapular incongruity associated with SSS. |
| Nijs et al. 2005 | 29 | NR | Physical exam test | 3 Tests: Lateral scapular slide test, measurement of distance from the medial border of the scapular to spinous process of T4, and measurement of distance between the posterior border of the acromion and the table when supine. | 2 of 3 tests had favourable interobserver reliability, but the clinical importance of the tests is questionable. |
| O'Connor et al. 2016 | 15 | 19.46 ± 0.63 | Clinical screening tool | Screening tool that evaluated scapular winging, lack of control when lifting/lowering the arm, and scapular symmetry. | This simple screening tool demonstrated high reliability in assessing scapular dyskinesis. |
| Park et al. 2013 | 89 | 19.2 | 3D CT | 3D CT was used to evaluate scapular positioning in patients with scapular dyskinesis. | 3D CT allowed precise quantification of a position associated with scapular dyskinesis. |
| Percy et al. 1988 | 14 | 33 (16-59) | Review of clinical findings | Clinical findings were reviewed of 14 patients with SSS. | The most common clinical signs were tenderness at the medial border of the scapula, palpable crepitus, and slightly audible snapping. |
| Shadmehr et al. 2016 | 50 | 42 ± 2.7 | Physical exam test | Modified lateral scapular slide test (LSST). | The modified LSST had good reliability and agreement properties to assess scapular position. No test position though had clinical utility as a diagnostic criterion. |
| Spiegl et al. 2015 | 26 | 36.8 (18-68) | MRI | MRI to assess scapular bony morphology. | Anterior angulation of the medial scapula in the axial plane was associated with SSS. |

**Appendix Table 2. Studies investigating the treatment of scapular dyskinesis and SSS.**

| **Study** | **Study Design** | **Patients** | **Mean Age** | **Mean Follow Up (months)** | **MINORS Score** | **Treatment Type** | **Main Outcomes** |
| --- | --- | --- | --- | --- | --- | --- | --- |
| Acar 2017 | Prospective Cohort | 35 | 36.2 ± 6.8 | 12 | 18 | Extracorporeal shock wave therapy (ESWT). | Low-energy ESWT showed good early-term results, however middle-energy ESWT demonstrated better early, mid, and late-term results |
| Acar et al. 2017 | RCT | 43 | 43.2 ± 5.6 | 6 | N/A | Extracorporeal shock wave therapy (ESWT) compared to steroid injection. | When compared to steroid injection, ESWT had better pain relief at later follow-up dates (3 and 6 months). |
| Blønd et al. 2014 | Case Series | 20 | 40 (19-68) | 34.8 (24-60) | 12 | Arthroscopic scapulothoracic bursectomy and partial resection of the superomedial angle of the scapula. | Procedure was successful in 18 of 20 patients. There was a significant improvement in the WORC index. |
| Carbone et al. 2014 | Case Series | 24 | 48 (26-65) | 12 | 11 | Rehabilitation protocol for 1.5 hours per week for one year, in patients with a chronic type III AC dislocation. | Scapular dyskinesis resolved in 18 of 23 patients. |
| Chang et al. 2009 | Prospective Cohort | 22 | 40.1 ± 9.5 | 3 | 10 | Steroid injections into the scapulothoracic bursa. | Steroid injection led to significant reduction in VAS pain scores at 1, 2, and 3 weeks post-treatment. |
| Chang et al. 2014 | RCT | 36 | 43.1 ± 10.1 | 3 | N/A | Compared intramuscular subscapularis and scapulothoracic bursa injections of corticosteroid with local anesthetic. | In patients with scapular pain, both injections into the subscapularis and scapulothoracic bursa provide equal symptomatic relief. |
| de Amorim et al. 2014 | RCT | 30 | 23 ± 4.2 | 2.5 | N/A | Compared global postural reeducation (GPR) to segmental stretching exercises. | Both were effective at improving upper extremity function, however GPR was superior in improving pain and quality of life. |
| Ermiş et al. 2012 | Case Series | 15 | 15.6 (6-29) | 164.4 (12-312) | 10 | Open resection of the superomedial angle of the scapula. | Mechanical symptoms of SSS were relieved in all patients. |
| Harper et al. 1999 | Case Series | 7 | 34 | 3 | 8 | Arthroscopic resection of the superomedial angle of the scapula. | 6 of 7 patients had successful management of SSS. |
| Lesprit et al. 2001 | Case Series | 13 | NR | 24 (12-50) | 7 | Open resection of the superomedial angle of the scapula. | 7 of 8 had favourable results after surgical treatment. |
| Lien et al. 2008 | Case Series | 12 | NR | 37.2 (24-60) | 12 | Arthroscopic scapulothoracic bursectomy and mini-open partial resection of the superomedial angle of the scapula. | Clinical resolution in 10 of 12 patients. Improved ASES and SST scores, with decreased pain scores. |
| Menge et al. 2016 | Case Series | 79 | 33 (12-65) | 40.8 (24-84) | 11 | Arthroscopic scapulothoracic bursectomy and partial resection of the superomedial angle of the scapula. | Improvement in ASES, QuickDASH, and SF-12. Treatment was successful in 64 of 72. |
| Menge et al. 2017 | Case Series | 74 | 33 (12-65) | NR | 10 | Arthroscopic scapulothoracic bursectomy and partial resection of the superomedial angle of the scapula. | Improvement in ASES, QuickDASH, and SF-12. Median patient satisfaction of 7 out of 10. |
| Merolla et al. 2010 | Case Series | 29 | 23 ± 4.2 | NR | 10 | Rehabilitation aimed at restoring scapular muscle balance. | Restoration of scapular muscle balance led to decreased pain and increased strength in overhead athletes with scapular dyskinesis. |
| Merolla et al. 2014 | Prospective Cohort | 10 | 24 ± 4.6 | 24 | 12 | Arthroscopic scapulothoracic bursectomy and selective partial resection of the superomedial angle of the scapula. | Improvements in WORC index, Constant-Murley Score, and SST. |
| Millett et al. 2012 | Case Series | 21 | NR | NR | 10 | Arthroscopic scapulothoracic bursectomy and partial resection of the superomedial angle of the scapula. | Improvement in ASES, SANE, and QuickDASH scores. 3 of 21 had to undergo revision for persistent pain. |
| Nicholson et al. 2002 | Case Series | 17 | 35 (19-53) | 30 (24-72) | 10 | Open scapulothoracic bursectomy. | Improvement in ASES and SST scores. All patients reported that painful crepitus had resolved. |
| Pavlik et al. 2003 | Case Series | 10 | 26.9 (16-40) | 11.5 (3-23) | 10 | Arthroscopic scapulothoracic bursectomy and partial resection of the superomedial angle of the scapula. | All patients had decreased or resolved crepitus. Pain scores were decreased and 9 of 10 patients were able to return to preoperative work. |
| Pearse et al. 2006 | Case Series | 13 | 35 (18-52) | 18.5 | 11 | Arthroscopic scapulothoracic bursectomy and partial resection of the superomedial angle of the scapula. | 9 of 13 reported an improvement in their symptoms. |
| Pekyavas and Ergun 2017 | RCT | 30 | 40.5 ± 12.5 | 2.5 | N/A | Compared home exercise program to virtual reality (VR) exergaming. | Both groups experienced significant pain reduction, however the VR exergaming group performed better in clinical tests for scapular dyskinesis. |
| Provencher et al. 2017 | Case Series | 46 | 25.5 (18-33) | 25.3 (24-30) | 13 | Open pectoralis minor release. | Improvement in ASES and SANE scores. Significant decrease in VAS pain scores. |
| Ross et al. 2009 | Case Series | 5 | 34 (24-41) | 12 (2-27) | 8 | Open resection of the superomedial angle of the scapula. | All 5 patients had resolution of their mechanical symptoms. |
| Sisto et al. 1986 | Case Series | 4 | 20.5 (20-24) | 12.3 (12-13) | 11 | Open scapulothoracic bursectomy. | All four professional pitchers returned to sport at their preoperative level. |
| Tashjian et al. 2013 | Case Series | 13 | 42 (21-62) | 27 (13-65) | 12 | Arthroscopic scapulothoracic bursectomy and partial resection of the superomedial angle of the scapula. | Improvement in SST scores and decreased VAS pain scores. 12 of 13 patients were satisfied. |
| Vastamaki and Vastamaki 2016 | Retrospective Cohort | 21 | 24.8 (12-46) | 230.9 (12-420) | 14 | Open resection of the superomedial angle of the scapula. | Both those treated operatively and nonoperatively had improved pain scores, however surgically treated patients improved faster and had presented with more pain to begin with. |
| Wood et al. 1989 | Case Series | 13 | 34 (24-46) | 36.2 (3-90) | 7 | Open resection of the superomedial angle of the scapula. | Complete relief of symptoms was achieved for all patients. |

**Appendix Table 3. Studies investigating post-traumatic scapular dyskinesis.**

| **Study** | **Patients (n)** | **Mean Age** | **Male/Female** | **Mean Follow Up (months)** | **Primary Injury** | **Number Developing Scapular Dyskinesis (%)** |
| --- | --- | --- | --- | --- | --- | --- |
| Shields et al. 2015 | 24 | 46 ± 17 | 16/8 | 20.4 ± 12 | Displaced Midshaft Clavicle Fracture | 1 of 12 (8%) treated surgically 8 of 12 (67%) treated nonoperatively |
| Gumina et al. 2009 | 34 | 47 (24-69) | 32/2 | 28 (12-36) | Type III Acromioclavicular Dislocation | 24 of 34 (70.6%) with conservative treatment |
| Murena et al. 2012 | 34 | 41.8 ± 10.2 | 33/1 | 82.7 ± 28.5 | Type III Acromioclavicular Dislocation | 4 of 34 (11.7%) following surgical treatment |

**Appendix Table 4. Cochrane Risk of Bias in RCTs.**

|  | **Random sequence generation** | **Allocation concealment** | **Blinding of participants & personnel** | **Blinding of outcome assessment** | **Incomplete outcome data** | **Selective reporting** | **Other Biases** |
| --- | --- | --- | --- | --- | --- | --- | --- |
| Acar et al. 2017 | L | L | H | H | L | L | U |
| Chang et al. 2014 | L | L | L | H | L | L | U |
| de Amorim et al. 2014 | L | L | H | H | L | L | U |
| Pekyavas and Ergun 2017 | L | L | H | H | L | L | H |
